# Assessment of Post-Operative Neurosensory Deficiency Following Le Fort I Maxillary Osteotomy and Its Impact on Patient Satisfaction: A Retrospective Clinical Cross-Sectional Study

**DOI:** 10.3390/jcm14041115

**Published:** 2025-02-09

**Authors:** Yasser S. Alali, Haya Dokhi Aldokhi, Rawan Ahmad Alayoub, Wajdi A. Mohammed (Bin), Sami Alshehri, Muath Alshayban

**Affiliations:** 1 Department of Oral and Maxillofacial Surgery, College of Dentistry, King Saud University, Riyadh 11545, Saudi Arabia; 2 Department of Family Dentistry, King Abdulaziz Medical City, Ministry of National Guard Health Affairs, Riyadh 12372, Saudi Arabia; hayaaldokhi@gmail.com; 3 Department of Pediatric Dentistry, Dar Al Uloom University, Ministry of Health, Riyadh 13314, Saudi Arabia; rawan.ahmad20@hotmail.com; 4 Department of Oral Medicine and Diagnostic Sciences, College of Dentistry, King Saud University, Riyadh 11545, Saudi Arabia; wbinmohammed@ksu.edu.sa; 5 Department of Biomedical Dental Sciences, College of Dentistry, Imam Abdulrahman Bin Faisal University, Dammam 31441, Saudi Arabia; smalshehri@iau.edu.sa; 6 Department of Restorative Dental Sciences, College of Dentistry, King Saud University, Riyadh 11545, Saudi Arabia; malshayban@ksu.edu.sa

**Keywords:** Le Fort 1 osteotomy, maxillary osteotomy, orthognathic surgery, neurosensory deficiency, patient satisfaction

## Abstract

**Background/Objectives**: Le Fort I maxillary osteotomy (LF1-MO) is associated with a risk of infraorbital nerve neurosensory deficiency (NSD). This study aimed to evaluate post-operative subjective numbness and objective NSD after LF1-MO and assess the impact of these outcomes on overall patient satisfaction. **Methods**: A retrospective cross-sectional study was conducted among adult LF1-MO patients, who were evaluated for treatment satisfaction using a 10-item patient satisfaction questionnaire. In addition, subjective and objective NSDs were assessed post-operatively for six months. Overall patient satisfaction was compared against different variables (patient age, sex, and type of LF1-MO) and NSD. The proportion of subjective and objective NSDs were statistically correlated and compared against these variables, assuming a 95% significance level (*p* < 0.05). **Results**: A total of 58 LF1-MO patients in the age range of 20–38 years (mean–29.79 ± 4.62 years) were included in this study. Most patients were females (n = 48; 82.8%) and aged 30 years and older (n = 32; 55.2%). The overall mean patient satisfaction score was 27.38 ± 3.94 (range 12–30), which did not significantly differ based on patient age or sex. Patients who had advanced LF1-MO had significantly higher satisfaction scores (28.27 ± 1.85) compared to those who had impaction (24.61 ± 7.34) (*p* < 0.05). Subjective numbness and an abnormal “Level A” response to objective neurosensory testing were associated with poor patient satisfaction. There was significant statistical correlation between subjective and objective NSDs (Spearman’s rho–0.441; *p* < 0.01). Based on a chi-squared test, patients undergoing maxillary setback (subjective–88.9%; objective–44.5%) had significantly higher NSDs (*p* < 0.05). **Conclusions**: Most patients reported satisfaction after LF1-MO, particularly among females, those aged 30 and older, and those without NSD. However, residual infraorbital NSDs persisted, with about two-thirds experiencing subjective numbness and 25% showing abnormal responses in “Level A” objective neurosensory tests six months post-operatively. Moreover, subjective numbness correlated with abnormal objective testing results, leading to lower patient satisfaction.

## 1. Introduction

Orthognathic surgery for correction of maxillary and mandibular skeletal malocclusion and dentofacial deformities is a routinely performed procedure in oral and maxillofacial surgery [1]. Common orthognathic surgical procedures include segmental osteotomies of the maxilla and mandible, Le Fort I osteotomy of the maxilla, and bilateral sagittal split osteotomy (BSSO) of the mandible. Other frequently performed procedures are midline maxillary osteotomy for palatal expansion, vertical ramus osteotomy, and genioplasty [2,3]. In the majority of patients who require simultaneous correction of maxillofacial skeletal deformities, it is common practice to perform bi-jaw or bi-maxillary osteotomies [4]. This typically involves a combination of the Le Fort I osteotomy, which addresses the upper jaw by repositioning the maxilla, and BSSO, which is utilized to correct the mandible [4,5]. These procedures are standard due to their effectiveness in achieving optimal alignment and functionality of the jaw structures, thereby improving both aesthetic outcomes and the patient’s overall oral function [2,6].

Le Fort I osteotomy of the maxilla is based on the principle of the midfacial fracture lines originally described by René Le Fort in 1901 [1,7]. Accordingly, Le Fort I osteotomy involves surgery for bony dysjunction of the inferior maxilla, including the teeth supporting the alveolar bone, hard palate, and the floor of the nose and maxillary sinus [1,2]. This is followed by repositioning the Le Fort I maxillary segment in three-dimensional planes based on orthognathic treatment objectives, which could either be advancement, impaction, or setback. In short, the Le Fort I osteotomy cuts extend bilaterally from the midline to the zygomatic buttresses and pterygomaxillary articulation. Additionally, osteotomies are performed on the nasal septum and lateral nasal walls to complete the procedure. Modifications to the procedure include a high-level Le Fort I osteotomy and combined orthognathic surgeries involving segmental or midline maxillary osteotomies [2,8].

Le Fort I maxillary osteotomy is one of the most widely used procedures and allows for the correction of dentofacial deformities in different dimensions, such as elongation, shortening, advancement, retrusion, and segmentation [9]. Although exact statistical estimates are not reported, Le Fort I osteotomies comprise nearly one-half to two-thirds of all orthognathic surgical procedures performed, either stand-alone or associated with mandibular osteotomies [4]. Le Fort I osteotomy is most commonly indicated for correcting and managing different types of skeletal malocclusion, including class II and III maxillary deformities [9]. In addition to being versatile, the skeletal correction based on Le Fort I osteotomy can be achieved with considerable ease, effectiveness, and reproducibility if proper preoperative and intraoperative preparations are taken. Despite all the advancements in surgical techniques and the psychomotor skills of surgeons, intraoperative complications are still encountered [10,11]. Frequently reported complications during Le Fort I osteotomies include improper osteotomy leading to bad splits, hemorrhage due to vascular injuries, nerve exposure and damage, dental injuries, and soft tissue injuries [10,11]. In addition, post-operative complications, such as residual malocclusion, dyspnea, temporomandibular disorders (TMD), malunion or nonunion of bone fractures, and paresthesia due to cranial nerve injuries, are also reported [12,13].

**Figure 1:**
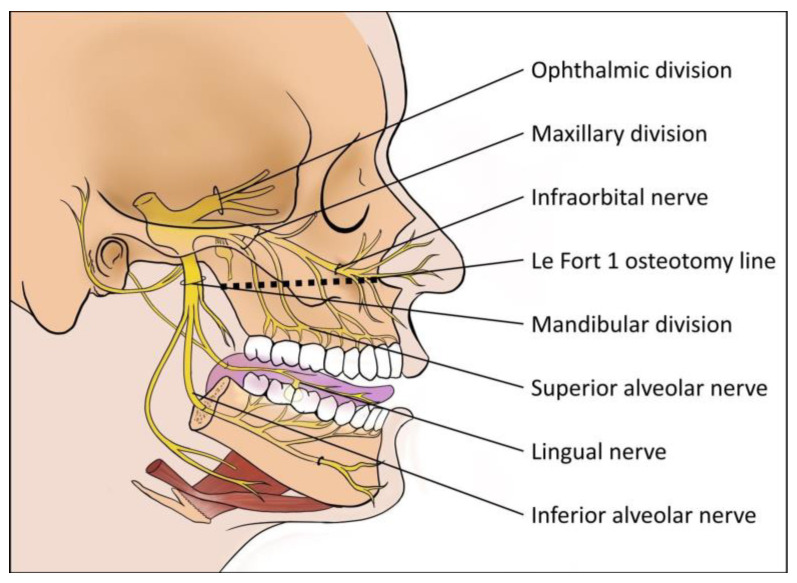
Sensory and cutaneous innervation of the face by the maxillary and mandibular nerve and its branches. Black dotted transverse line in the maxillary bone indicate the representative Le Fort I osteotomy cut.

Cranial nerve injury is one of the most serious complications that can occur after Le Fort I osteotomy, which may cause changes in the skin’s sensitivity over the infraorbital, lateral nasal, and upper lip regions [12]. Additionally, patients may report neurosensory deficiency in the mucosa of the upper lip, gingiva, and teeth after the osteotomy procedure [13,14]. The maxillary bone and its investing soft tissues, both intraoral and extraoral, are innervated by the maxillary branch of the fifth cranial (trigeminal) nerve [2,8,15]. The nature of the Le Fort I maxillary osteotomy cuts is such that they endanger the fine sensory nerve endings of the maxillary dentition and soft tissues, as shown in Figure 1 [16]. Although motor nerve injury and deficit following Le Fort I osteotomy are extremely rare, neurosensory deficiency and deficit are often always concomitant and show a longitudinal recovery pattern that could range from weeks to months [9]. This is further compounded by factors such as patient age, systemic status, and nature of the intraoperative maxillary manipulation [17]. In addition to being a major cause for concern among patients, loss of sensation following elective orthognathic surgery, such as Le Fort I osteotomy, is a major determinant of treatment satisfaction and post-operative quality of life [17].

Numerous studies have consistently highlighted a high degree of patient satisfaction following orthognathic surgery, with individuals primarily prioritizing aesthetic improvements and functional outcomes related to their jaw alignment and overall facial appearance [5,6,18]. While there is evidence from the literature indicating that neurosensory deficit can detrimentally affect patient satisfaction—particularly following orthognathic procedures—it is noteworthy that most of these findings are predominantly linked to mandibular osteotomies [17,19,20,21,22]. This research was specifically designed to draw attention to the significance of perceived and actual neurosensory deficiencies that may occur after maxillary Le Fort I osteotomies and to examine how these deficiencies influence overall patient satisfaction. Accordingly, this retrospective clinical cross-sectional study aims to evaluate post-operative subjective numbness and objective neurosensory deficiency in patients who underwent Le Fort I maxillary osteotomy and to assess the impact of these outcomes on overall patient satisfaction.

## 2. Materials and Methods

### 2.1. Study Design, Ethical Approval and Sampling

This retrospective clinical cross-sectional study was conducted in the Department of Oral and Maxillofacial Surgery, College of Dentistry and Dental University Hospital, King Saud University Medical City. Ethical approval for this study was obtained from the research ethics review committee and institutional review board (IRB) at King Saud University Medical City (IRB Approval No. E-22-6997, 20 October 2022). Informed consent was obtained from all the study participants for the use of their clinical, radiographic, and demographic data without disclosing identifying information.

The present study was conducted within a two-year time period beginning from January 2022 and ending in December 2023. The sample size for this study was estimated based on the previously reported percentage of longitudinal neurosensory deficiency following Le Fort I osteotomy (19.7% after 6 months) [9]. This study is based on the following assumptions, namely, statistical power—80%; confidence level—95%; and significance level—0.05 (*p* < 0.05 means statistically significant). Accordingly, the minimum sample size for an observational cross-sectional study was estimated to be 48 patients [23,24]. The sampling frame comprised all patients who underwent Le Fort I maxillary osteotomy for orthognathic surgical correction of maxillary skeletal malocclusion/deformity and reported for a continuous minimum follow-up of at least six months.

### 2.2. Inclusion and Exclusion Criteria

The main criteria for selecting patients for this study included being in good physical health (ASA1 or ASA2) without serious systemic illnesses and being adults aged between 18 and 40 years. Patients with a history of neuropathic diseases, psychiatric conditions, or those currently undergoing treatment for anxiety disorders were not eligible for inclusion.

Individuals with neurosensory deficiencies in the oral and maxillofacial regions, irrespective of whether these were primary or secondary to trauma or dental procedures, such as extractions and restorations, were excluded from this study.

To ensure consistent reporting of post-operative outcomes related to patient satisfaction, individuals who experienced significant complications following maxillary osteotomy, such as severe wound dehiscence, delayed healing, or infections, were also excluded from participation in this study.

### 2.3. Data Collection

Demographic data of the selected patients, namely patient age and sex, were collected from the patient records, along with information about the nature of Le Fort I maxillary osteotomy. In addition, patients were recalled for a follow-up visit and answered questions pertaining to subjective cutaneous numbness in the infraorbital and upper lip regions in the patient satisfaction questionnaire. While patients subjectively responded either as “yes” or “no” for the presence or absence of neurosensory deficiency, they were asked to answer a 10-item questionnaire to evaluate patient satisfaction following orthognathic surgery. The questionnaire was adapted from a previously reported study on patient satisfaction following orthognathic surgery in a middle-eastern population by AlKharafi et al. [5]. The original study had nine items as part of the questionnaire, which was modified to ten items in our study for ease of quantification (Table 1). The modified questionnaire was translated into the Arabic language (for the benefit of non-English speaking patients) and tested on a pilot sample of 15 volunteers for evaluating clarity and internal consistency. For each questionnaire item, patients responded their level of satisfaction, either as “Good”, “Fair”, or “Poor”, and it was mandatory for them to respond to all the items. The responses were graded on a three-point Likert scale (Good—3/Fair—2/Poor—1), translating to a total possible patient satisfaction score ranging between 10 and 30.

**Figure 2:**
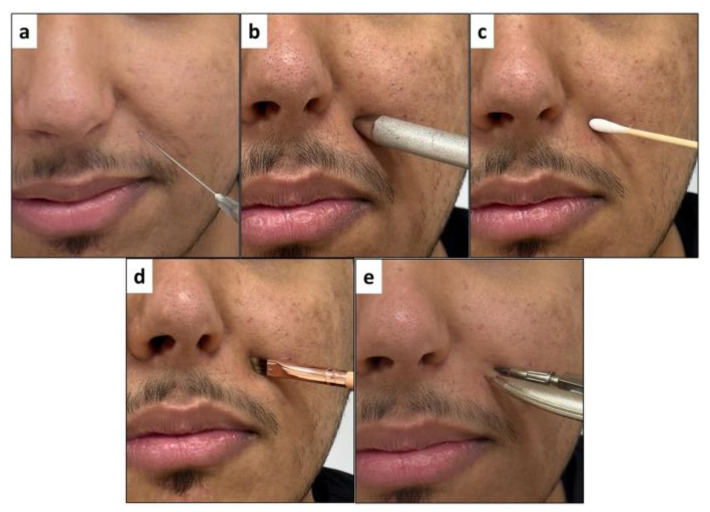
Objective testing for cutaneous neurosensory deficiency using the following stimuli: (**a**) pin-prick; (**b**) thermal (cold/heat); (**c**) static light-touch; (**d**) directional discrimination; and (**e**) two-point discrimination.

Objective testing for cutaneous neurosensory deficiency was performed by the same operator (Y.A.). It was focused on evaluating the neurosensory response to the following stimuli: pin-prick, thermal (cold/heat), static light-touch, directional discrimination, and two-point discrimination [20,25]. The armamentarium for the tests included a dental local anesthetic needle (pin-prick), dental explorer handle (thermal) exposed to heat and cold temperatures, cotton buds (static light-touch), painting brush (directional discrimination), and Boley gauge (two-point discrimination). The objective response for each level of testing method was quantified either as positive or negative. The methodology for each level of testing and a brief description for each method are shown in Figure 2 and described in Table 2, respectively.

### 2.4. Statistical Analysis

Collected data were tabulated on a spreadsheet software (MS-Excel, Microsoft Corporation, Redmond, WA, USA) and analyzed statistically using a statistical software package (IBM SPSS Statistics Ver. 21, IBM Corporation, Armonk, NY, USA). Descriptive statistics were calculated for all the independent variables (sex, age group, type of Le Fort I osteotomy, subjective numbness, and response to objective testing) and patient satisfaction score (dependent variable). Independent t-test and one-way ANOVA were used to compare the means of patient satisfaction score against the different independent variables. In addition, to compare the relationship between subjectively reported and objectively tested neurosensory deficiency and their association with the remaining independent variables (patient sex, age group, and type of Le Fort I osteotomy), Spearman’s rank correlation and Pearson’s chi-squared test of independence were conducted. Based on the assumption of a 95% significance level, *p*-values less than 0.05 (*p* < 0.05) were considered statistically significant for all tests.

## 3. Results

A total of 58 patients who underwent Le Fort I maxillary osteotomy within the sampling frame and fulfilled the inclusion criteria were included in this study. None of the patients reported any major intra- or post-operative complications. A majority of the patients were female (n = 48, 82.8%) and were in the age group of 30 years and older (n = 32, 55.2%). The overall mean age of the patients was 29.79 ± 4.62 years (range 20–38 years), with the mean age of patients aged 29 years and younger (n = 26, 44.8%) being 25.54 ± 2.76 years (range 20–29 years) and those in the age group of 30 years and older had a mean age of 33.25 ± 2.37 years (range 30–38 years). The mean satisfaction score of the patients was 27.38 ± 3.94 (range 12–30); differences in the mean values between different variable sub-groups, along with their statistical significance, are elucidated in Table 3.

### 3.1. Patient Satisfaction Following Le Fort I Osteotomy

In general, the mean patient satisfaction score was higher among female than male patients. Similarly, patients aged 30 years and older evinced greater satisfaction following Le Fort I maxillary orthognathic surgery than those who were 29 years old or younger. Nevertheless, the aforementioned differences were not statistically significant. In terms of the nature of maxillary movement following Le Fort I osteotomy, patients who underwent advancement of the maxilla showed greater mean satisfaction scores than those who underwent maxillary setback or impaction. The difference in mean satisfaction scores between patients with maxillary impaction and maxillary advancement was statistically significant (*p* < 0.05). Comparing the incidence of neurosensory deficit (both subjective and objective) following Le Fort I osteotomy with the patient satisfaction scores, there was no statistically significant difference between those who experienced neurosensory deficiency and those who did not. However, there was a greater satisfaction, as indicated by the higher patient satisfaction scores, among patients with no or minimal neurosensory issues (Table 3).

### 3.2. Neurosensory Deficit Following Le Fort I Osteotomy

Of the 58 patients included in this study, 36 patients (62.1%) reported subjective cutaneous numbness during the post-operative follow-up period. However, this varied upon objective evaluation, wherein only 14 patients (24.2%) responded negatively for Level A testing and showed positive responses to Level B testing (Table 3). This indicated the presence of only subjective paresthesia without actual neurosensory deficiency in 22 patients (37.9%). This was further confirmed through Spearman’s rank correlation between subjective numbness and Level A or B objective response to neurosensory testing, which showed a positive (Spearman’s rho–0.441) and highly significant correlation (*p* < 0.01) (Table 4).

Chi-squared tests to compare subjective numbness within the different variable sub-groups showed the greatest incidence among female patients (n = 30, 51.7%) and those aged 30 years or older (n = 20, 34.5%). While female patients had the highest percentage of response to objective neurosensory testing (Level A—62.1%, n = 36; Level B—20.7%, n = 12), there were more male patients who responded only to Level B testing (n = 10; 17.2%). Nevertheless, the differences in neurosensory response with respect to patient sex and age group were not statistically significant. On the contrary, the type of Le Fort I osteotomy showed statistically significant distribution of responses towards subjective numbness symptoms and objective neurosensory testing. While a statistically significant difference in reported subjective numbness was observed among patients who underwent Le Fort I maxillary setback, there was no statistical difference between those responding to Level A and B neurosensory testing. By contrast, patients who underwent maxillary impaction and advancement showed no significant differences in perceived numbness, while evincing a significantly higher response to “Level A” objective neurosensory testing. These findings indicate a greater potential for neurosensory deficit with maxillary setback (subjective—88.9%; objective—44.5%), than with impaction (subjective–40%; objective–nil) or advancement (subjective—53.3%; objective—20%) (Table 5).

## 4. Discussion

Le Fort I maxillary osteotomy is a frequently performed surgical procedure to correct various maxillary skeletal and dentofacial deformities [9]. Repositioning the maxilla following osteotomy through impaction, advancement, and setback enables three-dimensional correction of malocclusion, hypoplasia, hyperplasia, asymmetry, cleft lip, and palate-related deformities [1,2,8]. Favorable outcomes relating to esthetics, function, and enhancement in quality of life have resulted in Le Fort I maxillary osteotomy being reported as a surgical procedure with a high degree of patient satisfaction [26]. In addition to the perceived improvements in esthetics and function, the quality of pre-operative information about the surgical procedure and the likely benefits and risks is a significant determinant of patient satisfaction [27]. Cutaneous neurosensory deficiency following Le Fort I maxillary osteotomy is one such potential post-operative risk, which is often overlooked as consequential and something the patient has already consented to [17]. This is why the present study has endeavored to evaluate not only patient satisfaction after Le Fort I maxillary osteotomy but the percentage of neurosensory deficiency and the relationship between the two which, to the best of our knowledge, is its unique aspect. All the patients in this study underwent surgery for maxillary orthognathic surgery, and no major untoward complications were reported either intra- or post-operatively. The present sampling frame and outcome assessment were similar to the study reported by Alolayan and Leung, who evaluated longitudinal neurosensory recovery after Le Fort I osteotomy [9]. Even the patient population’s demographic distribution closely matched in terms of patient sex distribution and mean age, indicating a preference for orthognathic surgery among females and patients around 30 years of age [9].

Although statistically not significant, in our study, greater patient satisfaction following Le Fort I osteotomy was observed among females, patients aged 30 years and older, and patients who either experienced no subjective neurosensory deficiency or responded positively to Level A objective neurosensory testing (Table 3). However, patients who underwent maxillary advancement showed significantly higher satisfaction scores than those who underwent surgical impaction of the maxilla. The most pertinent aspect of patient satisfaction following orthognathic surgery remains the esthetic outcomes of treatment, which was evident in the present study, too [2,6]. Based on a recent review, Almasri et al. reported no significant differences in patient satisfaction after orthognathic surgery based on demographic factors, such as patient age and sex [6]. They reported greater satisfaction among patients undergoing bimaxillary orthognathic surgical correction for class III skeletal tendencies [6]. However, contrary to what was reported in the aforementioned review, the present study observed greater satisfaction scores among females and patients aged 30 years or older (Table 3). Although the present study did not individually evaluate satisfaction based on Le Fort I osteotomy performed as a part of bimaxillary surgery, significantly higher mean patient satisfaction scores were reported among patients who underwent maxillary advancement. This correlates to treatments performed for class III skeletal malocclusion and is coherent with what has been reported by Almasri et al. [6].

The most critical aspect of patient satisfaction scores obtained through this study was how subjective and objective neurosensory deficits impacted them. Although not statistically significant, poor satisfaction scores were observed among patients who reported subjective numbness and those who had abnormal responses to “Level A” objective neurosensory testing. Although studies from the literature have not reported the effect of neurosensory deficit on patient satisfaction after orthognathic surgery, AlKharafi et al. reported that patients were more likely to feel satisfied post-treatment when informed about potential surgical risks and discomfort pre-operatively [5,27]. As a part of the healthcare quality procedure followed in our hospital/center, all patients undergoing orthognathic surgery are informed about the risks of surgery, including neurosensory deficit, which could be prolonged in cases requiring severe skeletal correction. Le Fort I maxillary osteotomies are no exception to this informed consenting protocol and could explain why no statistically significant differences in patient satisfaction scores based on post-operative neurosensory deficit were observed in this study.

The close anatomic proximity of the infraorbital branch of maxillary nerve to the Le Fort I osteotomy cuts and the traction on the nerve caused by retraction and manipulation during surgery results in a significant neurosensory deficit in the region of cutaneous supply of the infraorbital nerve [16]. Patients often experience cutaneous numbness or paresthesia in the lower eyelid, lateral nose, upper lip, and cheek regions. While this could only be ipsilateral depending on the degree of traction over the infraorbital nerve, it is predominantly bilateral due to the three-dimensional anatomic nature of the Le Fort I maxillary orthognathic surgery [13,28]. Although neurosensory deficits are evidenced intraorally in the teeth, periodontium, and labial and alveolar mucosa, they are not as pronounced as the feeling of cutaneous numbness or paresthesia [17,28]. The most probable cause of cutaneous neurosensory deficit following Le Fort I maxillary osteotomy is neuropraxia due to traction on the infraorbital nerve and its branches [16]. Clinically, although it presents varying degrees of subjective and objective neurosensory loss, it is rarely permanent, and most patients regain normal cutaneous sensations between 6 and 12 months post-surgery [9,16,21]. On the contrary, axonotmesis or neurotmesis due to iatrogenic nerve injury would lead to permanent alteration or loss of sensation [12]. This can be ascertained through objective testing for neurosensory deficiency, as described in Table 2 [28,29,30].

Correlating the objective responses of Levels A, B, or C neurosensory testing to underlying neurological insult, Zuniga et al. classified nerve injury into five categories (normal, mild, moderate, severe, and complete) [30]. Wherein, normal indicates routine responses to Levels A, B, and C; mild indicates abnormal Level A response, but normal Level B and C response; moderate indicates abnormal Level A and B response, but normal level C response; severe indicates abnormal Level A and B response, in addition to hyperresponsiveness at Level C (hyperpathia and hyperalgesia); and complete indicates absent response to all levels of testing. While the mild and moderate categories indicate varying degrees of neuropraxia, the severe and complete categories of neurosensory deficit imply axonotmesis and neurotmesis, respectively [30].

In the present study, 36 of the 58 patients reported subjective numbness in the infraorbital region. Upon objective evaluation, 44 patients responded normally to Level A testing, and 14 had abnormal responses to Level A testing but provided a normal Level B response (Table 3). Neither did any patients report a complete loss of sensation, nor did they present elevated responses to testing, such as allodynia, hyperpathia, and hyperalgesia. Statistically, there was a significant positive correlation between subjective numbness and response to objective testing, with 14 patients reporting an abnormal Level A but normal Level B response having a definitive feeling of numbness (Table 4). Of the remaining 44 patients, 22 had a subjective sense of numbness while responding normally to Level A objective testing. This could be explained by mild neuropraxia, where patients experience an altered sensation (hypoesthesia or paresthesia), often misrepresented as numbness. However, objective testing yields expected results as there is no anatomic injury to the nerve. The aforementioned findings pertaining to neurosensory deficit six months post-Le Fort I osteotomy are coherent with those reported by Alolayan and Leung [9].

Comparing subjective and objective neurosensory deficit to the independent variables (patient sex, age group, and type of Le Fort I osteotomy) showed a significant relationship only with the nature of maxillary movement following osteotomy. While there was significantly greater subjective numbness reported by patients who underwent maxillary setback (88.9%), this correlated with an equivalent higher number of patients responding normally only to “Level B” objective neurosensory testing (44.5%). This could be attributed to the greater degree of intraoperative manipulation during a maxillary setback, as it requires down-fracturing the maxillary segment and removing bone distal to the maxillary third molar and the tuberosity [16]. In addition to the intraoperative difficulties, maxillary setback osteotomy could also negatively affect esthetics by retruding facial projection and compromising soft tissue support [31]. Nevertheless, posterior maxillary movement after osteotomy is indicated in cases where the setback distance is much less than a premolar width, usually gained through Le Fort I and anterior segmental osteotomy. Moreover, in patients whose premolar space has already been used to orthodontically correct crowding or those with congenitally missing premolars, maxillary setback is the only viable surgical option for posteriorly repositioning the maxilla [32]. Although the higher proportion of maxillary setback cases in the present study (n = 18, 31.1%) was greater than what is normally reported, all of these cases had clear indications for posterior maxillary movement over bimaxillary surgery. These included six patients who required minimal maxillary setback, nine patients who had their maxillary first premolars extracted and spaces closed orthodontically, and three patients whose periodontal compromise secondary to orthodontic treatment could have led to complications after anterior segmental osteotomy. Regarding neurosensory deficiency following Le Fort I osteotomy, it is often assumed that a maxillary impaction puts the infraorbital foramen and the infraorbital nerve at greater risk of iatrogenic injury [1]. However, the results of the present study indicate a significantly greater prevalence of neurosensory deficit following maxillary setback than with impaction (Table 5). It is, therefore, alluring to hypothesize that the choice of a maxillary setback following Le Fort I osteotomy should be carefully planned based on treatment needs and patient consent.

In the present study, subjective or objective neurosensory deficiency data were assessed in patients only at the six month follow-up and not on a longitudinal basis. This cross-sectional nature of data collection was a major limitation of the present study. However, assessing patient satisfaction and its relation to perceived neurosensory deficiency mandated us to define a time point for assessment, which was determined to be six months, based on a previously published study [9]. This study, by Alolayan and Leung [9], measured neurosensory recovery after Le Fort I osteotomy within a longitudinal timeframe from two weeks to two years post-operatively. According to Alolayan and Leung, neurosensory recovery follows a trajectory ranging from less than 20% at two weeks to more than 95% at two years, and it was greater than 80% by six months. Similar findings were observed at six months in our study, too. While objective quantification of neurological deficit in this study was performed by testing for different levels of neurosensory response, as described in Table 2 [20,25], no method was used for quantifying subjective numbness. Studies have reported and recommended using a visual analog scale (VAS) to assess subjective neurosensory symptoms [4,10,16]. Although this could be considered a minor limitation of the present study, it did not affect the evaluation of outcomes. Yet another limitation of the present study was the non-availability of data relating to confounding variables that may have contributed to nerve injury and subsequent neurosensory deficiency. In earlier reported studies, neurosensory deficiency after orthognathic surgery has been compared against several confounders, such as surgical time, surgeon’s experience level, maxillary segmentation, duration and length of nerve exposure, and nature of osteotomy instrumentation [10,17,20,22,33]. This was not considered in our study, as all the surgical procedures were performed by a team headed by the same consultant, following a defined protocol for Le Fort I osteotomy, and performed using reciprocating saw and rotary drill instrumentation. Furthermore, the cross-sectional nature of this study focused primarily on assessing the degree of neurosensory deficiency and how it impacted patient satisfaction, a significant determinant for clinical treatment success.

## 5. Conclusions

Based on the present study’s results, most patients indicated satisfaction with orthognathic surgical treatment following Le Fort I maxillary osteotomy. While this was as high as 91.3% (based on an overall mean satisfaction score of 27.38 ± 3.94, out of 30), in general, females, patients aged 30 years and older, and those who experienced no neurosensory deficit had higher patient satisfaction scores. Within the limitations of this study, it may also be concluded that Le Fort I maxillary osteotomy results in residual neurosensory deficiency in the cutaneous area of supply of the infraorbital nerve. For post-operative patients at six months, this could be as high as two-thirds of the patients reporting subjective numbness and about 25% of patients responding abnormally to “Level A” objective neurosensory testing.

According to our study, the nature of maxillary movement after Le Fort I osteotomy is an important determinant of patient satisfaction and neurosensory deficiency risk. While maxillary advancement was associated with significantly high patient satisfaction levels, orthognathic setback of the maxilla was related to a significantly high percentage of patients reporting subjective or objective neurosensory deficiency. Subjective numbness during the post-operative period may be a sign of underlying neurological deficit involving the infraorbital nerve as it statistically correlates with abnormal objective neurosensory testing results. It also proportionally translates to lower patient satisfaction following orthognathic surgical treatment.

The results of the present study indicate the necessity of informing and educating patients about the potential for neurosensory deficiency after Le Fort I maxillary osteotomy to set realistic patient expectations and achieve favorable post-operative quality-of-life outcomes. Moreover, further longitudinal, multicentric studies should be considered to ascertain the role of the multiple confounding factors capable of affecting neurological outcomes and, thereby, patient satisfaction after orthognathic surgery.

## Figures and Tables

**Table 1:** Patient Satisfaction Questionnaire, graded on a three-point scale (Good—3; Fair—2; Poor—1) to calculate an overall satisfaction score ranging from 10 to 30. (Adapted and modified from AlKharafi et al. [5]).

Questionnaire Items	Level of Satisfaction		
	Good	Fair	Poor
1. Esthetic appearance of the face			
2. Functioning of the jaw joints			
3. Ability to masticate food			
4. Clarity of speech and pronunciation of words			
5. Esthetic appearance of teeth			
6. Level of Self-confidence in the presence of others			
7. Level of Self-esteem to form new relationships			
8. Overall post-surgical recovery			
9. Overall satisfaction with orthodontic treatment			
10. Overall satisfaction with oral and maxillofacial surgical treatment			

**Table 2:** Objective testing for cutaneous neurosensory deficiency described in three-levels as Level A, Level B, and Level C depending on the method of testing, nerve fibers evaluated, and the procedure. An abnormal response to Level A testing necessitates further testing for Levels B and C [20,25].

Level of Testing	Testing Method	Nerve Fibers Evaluated	Procedure
Level A	Static light-touch	Larger A-α and A-β fibers (5–12 mm in diameter)	Using a cotton wisp or a painting brush, deliver 10 light strokes to the patient’s skin, and instruct the patient to indicate the direction of each stroke. Begin the assessment on the unaffected side, then proceed to the affected side. Record the number of correctly identified strokes; a normal score is 9 out of 10 correct responses.
	Direction discrimination		
	Two-point discrimination		Using a Boley gauge, patient is asked to identify the smallest distance at which they can differentiate two separate points. Compare the results between normal and affected sides. Normal values are 4–5 mm for the maxillary nerve and its branches.
	Thermal response (Hot/Cold)		Using a blunt metallic instrument exposed to moderate heat or cold, gently touch the skin until patient recognizes the stimulus as hot or cold. Begin the assessment on the unaffected side, then proceed to the affected side. Assess for allodynia (an abnormal pain response to a non-painful stimulus that stops once stimulus is removed).
Level B	Pin-prick	Smaller A-β fibers (4–8 mm in diameter)	Using a dental local anesthetic needle, gently prick the skin on the affected side. If there is no response, gradually increase the pressure until the skin is lightly indented. Assess for hyperpathia (an exaggerated response to a potentially painful stimulus, indicated by delayed-onset pain or increasing intensity with repeated stimuli). Also, evaluate for hyperalgesia (an abnormally increased sensitivity to pain identified by the patient experiencing pain on the affected side, which is out of proportion in comparison to the normal side).
Level C		Partially myelinated A-δ fibers and non-myelinated C fibers	

α—Alpha; β—Beta; δ—Delta.

**Table 3:** Descriptive statistics for the different variables and their respective sub-groups, along with mean patient satisfaction scores, and their statistical differences. (N = 58).

Variables and Sub-Groups	Frequency (%)	Patient Satisfaction Score	
		Mean (S.D.)	*p*-Value
Overall	58 (100%)	27.38 (3.94)	-
Sex			
Female	48 (82.8%)	27.58 (4.25)	0.384
Male	10 (17.2%)	26.41 (1.71)	
Age group			
29 years and younger	26 (44.8%)	26.69 (5.52)	0.058
30 years and older	32 (55.2%)	27.94 (1.81)	
Type of Le Fort I maxillary osteotomy			
Impaction	10 (17.2%)	24.61 (7.34) *	0.036
Advancement	30 (51.7%)	28.27 (1.85) *	
Setback	18 (31.1%)	27.44 (1.89)	
Subjective cutaneous numbness			
Yes	36 (62.1%)	26.72 (4.68)	0.343
No	22 (37.9%)	28.45 (1.92)	
Response to objective testing			
Level A	44 (75.8%)	27.45 (4.44)	0.154
Level B	14 (24.2%)	27.14 (1.71)	
Level C	-	-	

* indicates statistically significant difference.

**Table 4:** Spearman’s rank correlation between subjective cutaneous numbness and response to objective testing. (N = 58).

Subjective Cutaneous Numbness	Response to Objective Testing	
	Level A	Level B
Yes	22 (37.9%)	14 (24.2%)
No	22 (37.9%)	0

**Table 5:** Pearson’s chi-squared test of independence with adjusted residual for post hoc testing showing the statistical differences between the different variable sub-groups and neurosensory deficiency (subjective cutaneous numbness and response to objective testing (N = 58).

Independent Variables and Sub-Groups		Subjective Numbness			Response to Objective Testing		
		Yes	No	*p*-Value	Level A	Level B	*p*-Value
Sex	Female	30	18	0.882	36	12	0.737
	Male	6	4		8	2	
Age group	29 years and younger	16	10	0.940	22	4	0.160
	30 years and older	20	12		22	10	
Type of Le Fort I maxillary osteotomy	Impaction	4	6	0.014	10 *	0	0.023
	Advancement	16	14		24 *	6	
	Setback	16 *	2		10	8	

* indicates statistically significant difference.

## Data Availability

Study datasets shall be made available upon justifiable request to the corresponding author.

## Abbreviations

The following abbreviations are used in this manuscript:

LF1-MO	Le Fort 1 maxillary osteotomy
NSD	Neurosensory deficiency
IRB	Institutional review board
ASA	American society of anesthesiologists
